# Antifibrotic therapy to normalize the tumor microenvironment

**DOI:** 10.1186/s12967-020-02376-y

**Published:** 2020-05-20

**Authors:** Anette Hauge, Einar K. Rofstad

**Affiliations:** Group of Radiation Biology and Tumor Physiology, Department of Radiation Biology, Institute for Cancer Research, Oslo University Hospital, Oslo, Norway

**Keywords:** Antifibrotic therapy, Cancer-associated fibroblasts, Extracellular matrix, Tumor microenvironment, Profibrotic signaling pathways, Targeted treatments, Microenvironment normalization, Losartan

## Abstract

Most tumors develop abnormal fibrotic regions consisting of fibroblasts, immune cells, and a dense extracellular matrix (ECM) immersed in a viscous interstitial fluid, and an abundant fibrotic tumor microenvironment (TME) is associated with poor outcome of treatment. It has been hypothesized that the treatment of cancer may be improved by interventions aiming to normalize this TME. The approaches used in attempts to normalize the fibrotic TME can be categorized into three strategies of targeted antifibrotic therapy: targeting of components of the ECM, targeting of the producers of the ECM components—the activated cancer-associated fibroblasts (CAFs), and targeting of the signaling pathways activating CAFs. To target the ECM, enzymes against components of the ECM have been used, including collagenase, relaxin, hyaluronidase, and lyxyl oxidase. Targeting of CAFs have been investigated by using agents aiming to eliminate or reprogram CAFs. CAFs are activated primarily by transforming growth factor-β (TGF-β), hedgehog, or focal adhesion kinase signaling, and several agents have been used to target these signaling pathways, including angiotensin II receptor I blockers (e.g., losartan) to inhibit the TGF-β pathway. Taken together, these studies have revealed that antifibrotic therapy is a two-edged sword: while some studies suggest enhanced response to treatment after antifibrotic therapy, others suggest that antifibrotic therapy may lead to increased tumor growth, metastasis, and impaired outcome of treatment. There are several possible explanations of these conflicting observations. Most importantly, tumors contain different subpopulations of CAFs, and while some subpopulations may promote tumor growth and metastasis, others may inhibit malignant progression. Furthermore, the outcome of antifibrotic therapy may depend on stage of disease, duration of treatment, treatment-induced activation of alternative profibrotic signaling pathways, and treatment-induced recruitment of tumor-supporting immune cells. Nevertheless, losartan-induced suppression of TGF-β signaling appears to be a particularly promising strategy. Losartan is a widely prescribed antihypertensive drug and highly advantageous therapeutic effects have been observed after losartan treatment of pancreatic cancer. However, improved understanding of the mechanisms governing the development of fibrosis in tumors is needed before safe antifibrotic treatments can be established.

## Background

A solid tumor is an intricate ecosystem, consisting of a range of different cell types including parenchymal tumor cells, fibroblasts, endothelial cells, and immune cells, vasculature, and a scaffolding extracellular matrix (ECM)—all immersed in interstitial fluid. The various constituents interact closely with each other, creating a tumor microenvironment (TME) which is physiologically and structurally different from that in normal tissues. Common abnormal characteristics include hypoxia, low extracellular pH, nutrient deprivation, high interstitial fluid pressure (IFP), and a stiff and compact ECM—conditions known to promote tumor progression and impair the effect of treatment [1–4]. Hence, an increasing number of anticancer strategies aim to normalize the TME in order to potentiate established cancer therapies, like chemotherapy and radiation therapy, and achieve better tumor control [5–9].

Fibrosis (i.e., excess deposition of ECM components producing a fibrous connective tissue) is a critical feature of the TME in many solid tumors. Such thickening and scarring of connective tissue normally occur as a reparative response to injury or tissue damage, and is essential during the course of wound healing. In cancerous tissue, however, the process of fibrosis tends to be permanently activated, and accordingly, tumors have been described as “wounds that do not heal” [10]. Although the underlying mechanisms are not yet fully understood, the significance of tumor fibrosis and a dense ECM for cancer behavior—and thus the management of cancer patients—is now being increasingly acknowledged [11].

A major implication of the fibrotic TME is the increased solid stress experienced by cancer cells and other components of the tumor tissue [8, 12, 13]. In particular, excessive production of ECM molecules may lead to compression of blood vessels and significantly diminished perfusion and tumor oxygenation. Also, compressed vessels imply decreased supply of therapeutic agents to the tumor. Because certain important ECM molecules [e.g., hyaluronan (hyaluronic acid)] bind water, the abundance of such compounds has further been associated with elevated IFP [14]. Thus, a compact ECM does not only hinder the vascular transport of therapeutic molecules; it also affects the transfer of drugs across the vessel wall, as high IFP lowers the pressure gradients required for extravasation of drugs into the interstitial space [15–17]. Moreover, the transport of drugs through the interstitium is hampered by the ECM fibers themselves, acting as physical barriers to macromolecular movement [18, 19]. Finally, it is crucial to notice that the ECM is a highly dynamic network, whose structure and mechanical properties change over time. As such, it interferes with numerous molecular signaling pathways within and between the tumor cells, and consequently—other than affecting the tumor supply of blood, oxygen, and therapeutic agents—an abnormal ECM leads to altered molecular signaling in the TME [11]. Increased ECM stiffness has for instance been coupled with mechanical activation of signaling pathways that promote the survival and metastatic spread of cancer cells [20].

Not surprisingly, high expression of ECM molecules, such as collagen and hyaluronan, has been associated with poor outcome for patients with several types of cancer [21, 22]. As a consequence, it has been hypothesized that antifibrotic therapy (i.e., therapy aimed at reducing the amount of fibrosis) may be advantageous to cancer patients. Potential benefits include decreased solid stress and IFP, improved perfusion and tumor oxygenation, and a normalized TME resembling the microenvironment of corresponding normal tissues. Accordingly, antifibrotic therapy could allow for enhanced delivery and effect of anticancer agents. Various antifibrotic treatment strategies against solid tumors are outlined in this review, along with pivotal preclinical and clinical findings related to each approach. We also discuss the lessons learned from these studies, and provide some concluding remarks on the future prospects of this intriguing therapeutic concept.

## Antifibrotic treatment strategies in cancer

In tumors as in normal tissues, components of the ECM are produced mainly by fibroblasts, which are the most common connective tissue cells in humans and animals. The fibroblasts within a tumor are usually termed cancer-associated fibroblasts (CAFs). Only CAFs that are activated produce ECM compounds, and activation and proliferation of CAFs occur in response to soluble signaling molecules secreted by several cell types including immune cells, blood platelets, and cancer cells. The most essential and well characterized signaling pathway in this matter is the transforming growth factor-β (TGF-β) pathway. Other important signaling pathways include the hedgehog (Hh) pathway, the connective tissue growth factor (CTGF) pathway, and the platelet-derived growth factor (PDGF) pathway [23–25].

**Fig. 1:**
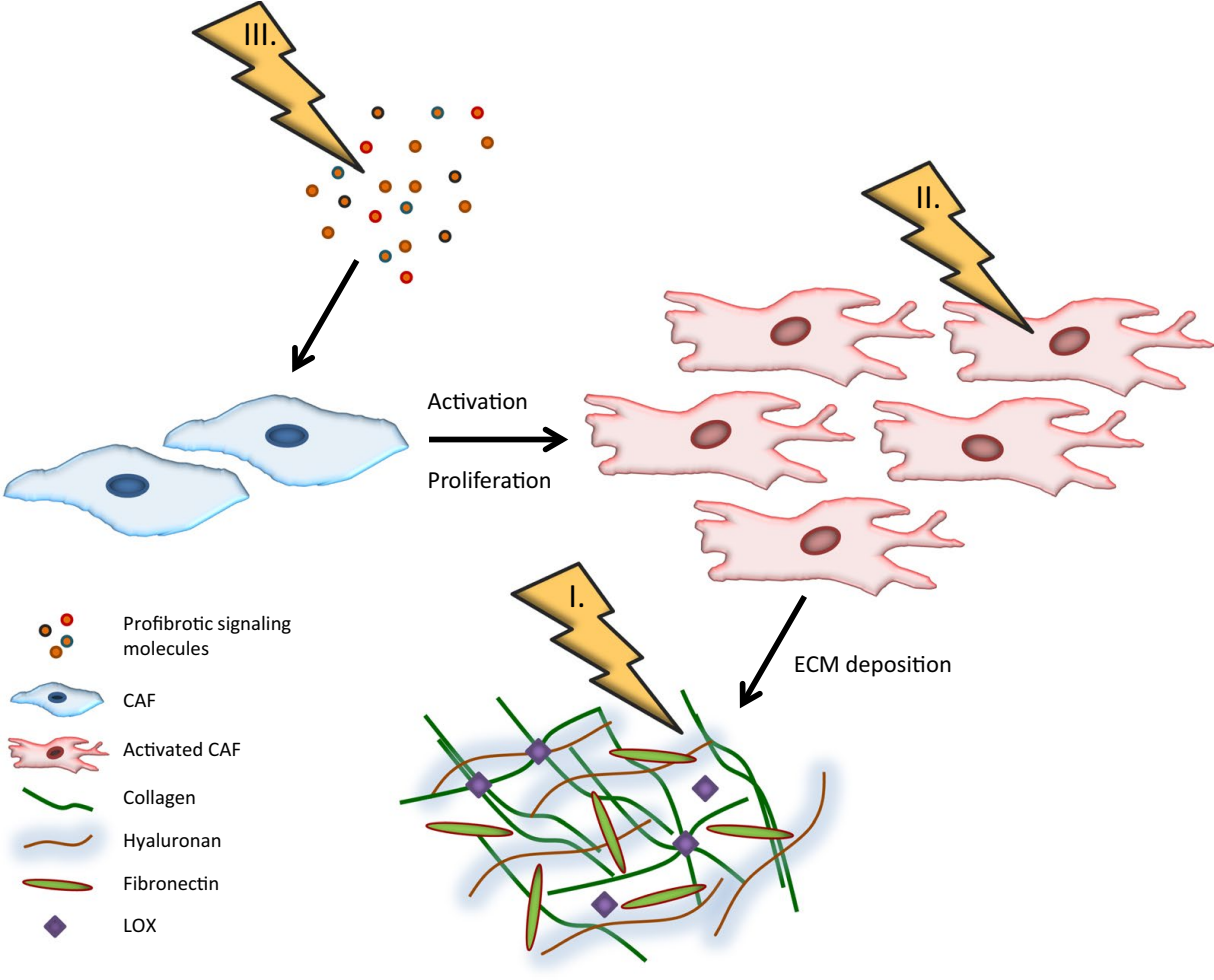
Treatment strategies against tumor fibrosis. Three main approaches of antifibrotic cancer therapy are currently being investigated: (I) targeting the extracellular matrix (ECM), (II) targeting cancer-associated fibroblasts (CAFs), and (III) targeting profibrotic signaling pathways. Collagen, hyaluronan, and fibronectin are major constituents of the extracellular matrix. Lysyl oxidase (LOX) is a major contributor to the stiffness of the tumor stroma

On the basis of this fibrotic “machinery”, reported strategies against tumor fibrosis can be categorized into three main approaches of antifibrotic therapy: direct targeting of the ECM, targeting of CAFs, and targeting of upstream profibrotic signaling (Fig. 1).

## Targeting the ECM

Direct targeting and depletion of ECM molecules, or alternatively, targeting of compounds of importance for the organization and cross-linking of ECM molecules, is one of the approaches being used in attempts to combat tumor fibrosis. Among essential ECM constituents are fibrous matrix proteins [e.g., collagen and elastin, glycosaminoglycans (e.g., hyaluronan), and various proteoglycans] and different cross-linking molecules and enzymes. Collagen is the most abundant protein in the ECM, and collagen and hyaluronan have been identified as main determinants of the transport of drugs and other molecules between the cells in a tumor [26–34]. Therefore, most antifibrotic strategies aiming to target the ECM directly have been directed at either of these two components. Different types of the enzyme collagenase, as well as the protein relaxin, have been tested for the breakdown of collagen fibers [35–39], and hyaluronidase has been used to cleave the hyaluronan polymer [40–42]. Several such matrix-depleting agents have improved the intratumoral distribution and efficacy of anticancer drugs in preclinical settings [43–51]. Nevertheless, this kind of treatment has also been associated with unacceptable normal tissue toxicity and increased risk of tumor progression [11, 52, 53].

An ECM-targeted drug of particular interest is the PEGylated recombinant human hyaluronidase PEGPH20 [54–60]. In pancreatic mouse tumors, treatment with PEGPH20 resulted in ablation of hyaluronan and lowering of IFP [61], although it should be noticed that untreated pancreatic tumors were reported to have strikingly high IFP values in this investigation [62, 63]. Even so, the remodeling of the tumor stroma appeared to be permanent and resulted in increased animal survival when combined with gemcitabine treatment [61]. A phase II clinical study in pancreatic cancer patients demonstrated that patients treated with PEGPH20 in addition to chemotherapy experienced increased progression-free survival as compared to patients treated with chemotherapy alone [64]. Further, the difference between the two treatment groups was more pronounced for a subset of patients having hyaluronan-rich tumors, in which the combined treatment resulted in a four-months delay in disease progression [64]. A phase III clinical study on adding PEGPH20 to nab-paclitaxel and gemcitabine was then initiated in patients with hyaluronan-rich pancreatic ductal adenocarcinoma (PDAC) [65]. Recently, it was revealed that the combined treatment failed to show an improvement in overall survival, duration of response, or progression-free survival versus gemcitabine and nab-paclitaxel alone, and Halozyme Therapeutics notified that the clinical development of PEGPH20 will be discontinued.

Studies investigating the structuring of the ECM as potential target are receiving increasing attention, and the enzyme lysyl oxidase (LOX) is of particular interest in this regard. LOX is a major contributor to the stiffness of the tumor stroma, as it up-regulates cross-linking of collagen fibers as well as cross-linking of collagen and other ECM components [4, 66–69]. Moreover, LOX is highly expressed under hypoxic conditions and has been identified as a driver of metastasis [70–80]. Inhibition of LOX combined with gemcitabine treatment in PDAC mouse models resulted in stromal alterations including reduced fibrillar collagen, suppression of metastasis, and extended disease-free survival of mice with early-stage tumors [81]. The small molecule LOX inhibitor PXS‐5505A (Pharmaxis Ltd), initially aimed at patients with myelofibrosis and pancreatic cancer, is currently being assessed in a clinical phase I study [82, 83].

## Targeting CAFs

**Fig. 2:**
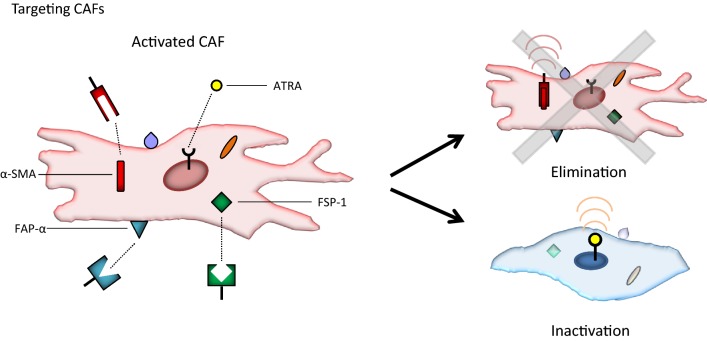
Strategies for targeting cancer-associated fibroblasts (CAFs). CAF populations can be eliminated by targeting molecular markers such as α-smooth muscle actin (α-SMA), fibroblast-specific protein-1 (FSP-1), and fibroblast activation protein-α (FAP-α). Alternatively, CAFs can be reprogrammed and inactivated by treatment with all-trans retinoic acid (ATRA), a metabolite of vitamin A that binds to a receptor in the nucleus of CAFs

Another antifibrotic strategy under investigation aims at targeting the producers of the ECM—the CAFs (Fig. 2). The CAFs interact closely with cancer cells in solid tumors and have been shown to serve multiple and diverse roles in cancer progression. Apart from depositing excess amounts of collagen fibers, hyaluronan, and other ECM constituents, CAFs are involved in angiogenesis, in creating an immunosuppressive TME that supports tumor growth, and in promoting metastasis [84–87]. One approach to identify and eliminate CAF populations is to exploit their expression of molecular markers such as α-smooth muscle actin (α-SMA), fibroblast-specific protein-1 (FSP-1), and fibroblast activation protein-α (FAP-α). This was tested by Özdemir et al. [88], who generated genetically modified mouse models of PDAC in which they could selectively reduce the number of α-SMA+ CAFs by pharmacological treatment. The aim was to interrogate the functional contribution of CAFs to PDAC initiation and development, and when eliminating activated CAFs, extensive ECM remodeling, a reduction in collagen content, and decreased tumor stiffness were observed. Nevertheless, the mice in lack of these CAFs also showed more aggressive tumors, immunosuppression, and reduced survival, suggesting a highly complex role of CAFs in cancer progression.

On a general basis, antifibrotic treatments that use targeting of CAFs as a strategy have faced significant obstacles. A plethora of molecular markers has been used to detect CAFs, but unique markers or specific markers that are expressed in all CAFs have not been identified [11]. The poor specificity restrains direct depletion of CAFs via molecular markers, and targeting CAFs without damaging normal tissue remains a challenge.

Furthermore, there is large heterogeneity among the CAFs in a tumor, and the causes and consequences of this heterogeneity are poorly understood. Differences among CAFs in the expression level and distribution of frequently used molecular markers suggest that there are different subpopulations of CAFs in tumors [89]. Several CAF subpopulations may co-exist in a single tumor, and different tumors may not necessarily have the same CAF subpopulations, not even tumors of the same histological category. Tumors show different subpopulations of CAFs primarily because CAFs may originate from different progenitor cells, including stellate cells, fibroblasts, fibrocytes, endothelial cells, and mesenchymal stem cells and, in addition, CAFs originating from the same cellular source may transdifferentiate into distinctly different subtypes depending on the juxtacrine and paracrine microenvironment [90–98]. Different subpopulations of CAFs may have dissimilar functions in tumors, and certain CAF subpopulations may serve a protective rather than a tumor-promoting role, as indicated by the findings of Özdemir et al. [88]. Consequently, antifibrotic treatments that inactivate or modulate CAFs may have unpredictable effects on tumor aggressiveness and require thorough evaluation before being used clinically.

Yet, along with improved understanding of CAF biology and dynamics, the enthusiasm for CAF-targeted therapies is currently growing and several promising preclinical studies have been reported [99–108]. For instance, instead of depleting CAFs, treatment with all-trans retinoic acid (ATRA; also known as tretinoin)—a metabolite of vitamin A—has been shown to reprogram activated CAFs and make them more quiescent [109]. This is achieved by ATRA binding to a receptor in the CAF nucleus, potentially leading to suppression of ECM remodeling and inhibition of cancer cell invasion [109]. Results are awaited from clinical phase I studies on reprogramming CAFs in pancreatic cancer using ATRA in combination with chemotherapy [110, 111].

## Targeting profibrotic signaling pathways

**Fig. 3:**
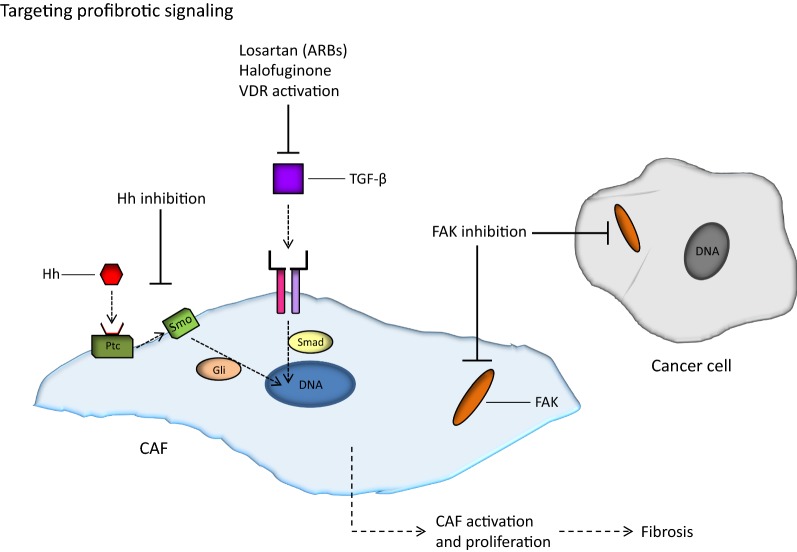
Strategies for targeting profibrotic signaling pathways. The two most important profibrotic signaling pathways are the hedgehog (Hh) pathway and the transforming growth factor-β (TGF-β) pathway. Binding of the Hh ligand to its receptor Patched (Ptc) enables the transmembrane protein Smoothened to activate the Gli transcription factors, ultimately leading to proliferation of cancer-associated fibroblasts (CAFs) and deposition of extracellular matrix components. Similarly, activation of the TGF-β pathway, mediated by the Smad proteins, activates CAFs and may cause tumor fibrosis. Several inhibitors of the Hh pathway have been developed, the most interesting being the Smoothened inhibitor IPI-926. The TGF-β pathway can be inhibited by angiotensin II receptor I blockers (ARBs) such as losartan, by halofuginone (a derivative of febrifugine), and by activating vitamin D receptor (VDR) signaling. Furthermore, high focal adhesion kinase (FAK) signaling in CAFs and parenchymal cancer cells may advance the formation of a fibrotic tumor microenvironment, and the FAK inhibitor defactinib is an efficient inhibitor of this pathway

Other than CAFs and the ECM, upstream signaling pathways that ultimately activate CAFs or otherwise promote the production of ECM constituents are potential targets for antifibrotic therapy (Fig. 3). This antifibrotic approach has been the focus of much attention in recent years, and several studies have been reported on interfering with the TGF-β or Hh signaling pathway in particular [112–118]. For instance, inhibition of TGF-β signaling via the angiotensin system has been successfully tested [119–123]. The peptide angiotensin II promotes fibrosis by binding to its receptor (angiotensin II receptor I) and thereby increasing the concentration of thrombospondin-1—a major activator of TGF-β. Angiotensin II receptor blockers (ARBs) may prevent this signaling cascade and cause suppression of active TGF-β levels and other profibrotic signals. As clinically approved drugs for the indication of hypertension, ARBs like losartan are of particular interest for this purpose, and the first use of losartan as an antifibrotic agent in tumors was described by Diop-Frimpong et al. [119] in 2011. Subsequent preclinical experiments with breast and pancreatic cancer models showed losartan to reduce the amount of both collagen and hyaluronan intratumorally, resulting in decompression of tumor vessels and significantly improved perfusion [120]. Furthermore, less hypoxia, enhanced delivery of chemotherapy, and improved overall survival were observed after losartan treatment—both for mice with breast tumors and mice with pancreatic tumors [120].

The promising preclinical reports on repurposing losartan as an anticancer agent, together with supporting retrospective clinical data, led to a phase II study being initiated for the treatment of locally advanced pancreatic cancer. After eight cycles of FOLFIRINOX chemotherapy and losartan, followed by chemotherapy and radiation therapy, 69% of the patients included in the study were eligible for surgical tumor resection [121], as compared to a resection rate of 26% found in a meta-analysis of twelve previous studies, in which locally advanced pancreatic cancer patients received chemotherapy and/or radiotherapy without losartan [122]. Nevertheless, more individualized chemoradiotherapy as well as the use of proton beam radiation in the losartan study may also have contributed to these improved resection rates. Interestingly, the use of angiotensin system inhibitors such as losartan has further been associated with enhanced activation of the immune system, and pancreatic cancer patients are now being recruited to a larger multicenter randomized phase II study on combining chemoradiotherapy and losartan with immunotherapy [123].

Halofuginone—a synthetic derivative of the antimalarial and antiparasitic agent febrifugine—is another potent antifibrotic agent blocking TGF-β signaling [124–126]. Due to its multifaceted antitumor effects in preclinical experiments, a phase I study of halofuginone was performed in patients with solid tumors refractory to standard therapy, leading to recommended doses for phase II studies [127]. Recent experiments in an autochthonous mouse model of PDAC demonstrated halofuginone to effectively remove biophysical barriers to drug delivery, and to increase the infiltration of antitumor immune cells, warranting further exploration of halofuginone as part of combination treatments against PDAC [22].

Although mostly quiescent in adult tissues, the Hh signaling pathway is hyperactivated in many solid tumors, and excessive Hh signaling promotes the generation of a fibrous interstitium and stimulates tumor growth [115, 118]. More specifically, binding of the Hh ligand to its receptor on the surface of CAFs enables the transmembrane protein Smoothened—which is otherwise repressed—to activate the Gli family of transcription factors, ultimately leading to CAF proliferation and deposition of ECM components [128–130]. Numerous inhibitors of the Hh pathway have been studied and are in varying stages of clinical development, following studies like the game-changing work of Olive et al. [131] in 2009 on the treatment of pancreatic mouse tumors with the Smoothened inhibitor IPI-926. Daily administration of IPI-926 for 8‒12 days caused depletion of collagen fibers, along with a transient increase in the density of blood vessels, both of which contributed to increased intratumoral concentration of gemcitabine and a transient stabilization of the tumor tissue. Furthermore, mice treated with IPI-926 and gemcitabine in combination had fewer liver metastases and extended survival as compared to control mice or mice treated with IPI-926 or gemcitabine alone [131].

In spite of encouraging findings in animal models of several cancer types, patient trials on Hh inhibition plus chemotherapy have yielded conflicting results [115–118]. Whereas robust antitumor activity and acceptable safety were reported after a phase I study on pancreatic cancer, other studies have failed to improve patient outcome, or even demonstrated more rapid disease progression and decreased survival for the combination regimen as compared to chemotherapy and placebo [132, 133]. Preclinical experiments intending to explain these contradictory findings revealed that long-term inhibition of the Hh pathway may result in more aggressive disease [134–137]. Accordingly, it was postulated that certain elements of the tumor stroma may act to restrain rather than support tumor growth, and moreover, that long-term antifibrotic therapy may fuel tumor progression [134].

Numerous profibrotic signaling pathways exist, and in addition to the angiotensin system and Hh signaling, vitamin D receptor (VDR) signaling and focal adhesion kinase (FAK) signaling have received significant attention [138, 139]. Activation of VDR by vitamin D or a vitamin D analogue interferes with TGF-β signaling and renders CAFs less active, and VDR-mediated manipulation of the tumor stroma has shown promise in enhancing the efficacy of pancreatic cancer therapy [138]. Also, because high FAK activity in cancer cells advances the formation of a fibrotic and immunosuppressive TME, FAK inhibition combined with immunotherapy—found to double the survival of mice with pancreatic tumors—represents an intriguing anticancer strategy [139]. At present, pancreatic cancer patients, as well as patients with mesothelioma, non-small-cell lung cancer, and other advanced solid tumors, are being treated with the FAK inhibitor defactinib in combination with the programmed cell death protein-1 (PD-1) inhibitor pembrolizumab in early phase clinical trials [140, 141].

## Lessons learned and points to consider

As justified by a great number of preclinical studies, therapeutic interventions with the intention to combat tumor fibrosis are currently under clinical evaluation. Nevertheless, in the wake of stirring reports of potential detrimental outcomes of certain antifibrotic approaches, questions have been raised about the relative benefit or harm of such therapy. As for the lessons learned thus far, one of the most important relates to the heterogeneity and dynamics of CAFs. Substantial heterogeneity has been demonstrated among the CAFs in a solid tumor, and various subpopulations may influence tumor development and progression differently [142–144]. Thus, while some CAF populations promote tumor growth, others may prevent malignant progression [145]. In consequence, antifibrotic therapy that somehow affects the number or properties of CAFs must be given with caution. Furthermore, as the role of CAFs may evolve with time and disease level, it is plausible that the response of a patient to CAF-targeting strategies will depend on the stage of the patient’s disease. Tumor stage-dependent effects would also be expected for several agents used to manipulate the ECM, since many such agents (e.g., LOX inhibitors) block only progressive ECM remodeling, and do not reverse previous activity. Therefore, advanced tumors with a well-established ECM may not respond to these medications.

Another critical issue is that of the duration of antifibrotic therapy. Studies have shown that long-term administration of antifibrotic agents may be unwise, as beneficial short-term effects (e.g., increased drug delivery) may be overcome by detrimental long-term effects (e.g., accelerated tumor growth) [134]. In fact, chronic, systematic depletion of fibrosis has been linked to increased metastatic ability and elevated presence of tumor-supporting immune cells [88, 134]. Also, when targeting profibrotic signaling pathways for a longer time period, alternative signaling pathways may be activated to restore the fibrous tumor stroma and cause treatment resistance [134]. This could explain the transitory effects observed after certain antifibrotic therapies, like inhibition of Hh signaling. In addition, it is important to recognize that the conditions within the tumor stroma, such as the expression level of CAF genes, may change during a course of treatment [11, 146].

Finally, CAFs and many profibrotic signaling molecules (e.g., Hh and TGF-β) exert multiple physiological functions and affect several cell types. Hence, interventions targeting these entities could cause bystander effects on other stromal compartments as well as adverse side effects. Also in this regard, short-term and pulsed administration of antifibrotic therapy—allowing fine-tuned manipulation of the tumor stroma as compared to long-term therapeutic intervention—is likely to be preferable, and to result in manageable toxicity [146].

## Conclusions and future perspectives

Preclinical studies and clinical investigations have evidenced that great care must be taken when developing antifibrotic strategies for normalizing the TME and, hence, improving the outcome of established cancer treatments. There are, nevertheless, strategies holding better promise than others, of which suppression of TGF-β signaling (e.g., induced by losartan) deserves special attention. Highly advantageous effects have been observed after losartan treatment [121, 123], and in addition, this drug is considered safe, it is relatively inexpensive, and it is already widely prescribed as an antihypertensive agent [147, 148]. In contrast, routine clinical use of treatments targeting the Hh pathway lies further up the road, as inhibition of Hh signaling has provided conflicting findings both in animal models and patients, despite it being crosstalk between the TGF-β and Hh pathways in cancerous tissue [118, 149]. Also therapeutic strategies aiming to degrade and/or destabilize the ECM directly as well as treatments targeting CAFs, such as ATRA treatment, have to be investigated further before their clinical potential can be assessed.

The increasing number of studies assessing antifibrotic cancer therapy have one conclusion in common: the microenvironment of solid tumors is extremely complex and greatly influences therapeutic resistance. Accordingly, there is an urgent need for enhanced understanding of the physicochemical and molecular mechanisms governing the TME. Furthermore, in order to identify subgroups of patients that might benefit from specific antifibrotic approaches (i.e., personalized antifibrotic therapy), improved methods for thorough characterization of the heterogeneous tumor stroma are required.

Whereas time will show whether fibrosis-targeting strategies will translate into improved outcome of patients with advanced solid tumors, recognizing the inherent limitations of antifibrotic therapy is crucial. For instance, despite successful manipulation of the ECM, severe gradients in intratumoral drug concentration and nutrient supply may still be present due to features like the abnormal and partly dysfunctional tumor vasculature. In other words, one may still have tumor regions in which the cells are more or less resistant to treatment because they are hypoxic, poorly nourished, and slowly proliferating or because they are not even exposed to the drug in question. Consequently, and as a result of the intricate interactions between different elements of the tumor stroma, combination therapies may be the future—not just antifibrotic therapy and conventional chemoradiotherapy, but also antifibrotic therapy combined with immunotherapy and/or antiangiogenic therapy.

## Data Availability

Not applicable.

## Abbreviations

ARB: Angiotensin II receptor blocker
ATRA: All-trans retinoic acid
CAF: Cancer-associated fibroblast
CTGF: Connective tissue growth factor
ECM: Extracellular matrix
FAK: Focal adhesion kinase
FAP-α: Fibroblast activation protein-α
FSP-1: Fibroblast-specific protein-1
FOLFIRINOX: Folinic acid + fluorouracil + irinotecan + oxaliplatin
Hh: Hedgehog
IFP: Interstitial fluid pressure
LOX: Lysyl oxidase
PD-1: Programmed cell death protein-1
PDAC: Pancreatic ductal adenocarcinoma
PDGF: Platelet-derived growth factor
PEGPH20: PEGylated recombinant human hyaluronidase
α-SMA: α-smooth muscle actin
TGF-β: Transforming growth factor-β
TME: Tumor microenvironment
VDR: Vitamin D receptor
